# 3Disease Browser: A Web server for integrating 3D genome and disease-associated chromosome rearrangement data

**DOI:** 10.1038/srep34651

**Published:** 2016-10-13

**Authors:** Ruifeng Li, Yifang Liu, Tingting Li, Cheng Li

**Affiliations:** 1 Peking-Tsinghua Center for Life Sciences, Academy for Advanced Interdisciplinary Studies; School of Life Sciences, Peking University, Beijing, China; 2 School of Life Sciences, Tsinghua University, Beijing, China; 3 Center for Statistical Science; Center for Bioinformatics, Peking University, Beijing, China

## Abstract

Chromosomal rearrangement (CR) events have been implicated in many tumor and non-tumor human diseases. CR events lead to their associated diseases by disrupting gene and protein structures. Also, they can lead to diseases through changes in chromosomal 3D structure and gene expression. In this study, we search for CR-associated diseases potentially caused by chromosomal 3D structure alteration by integrating Hi-C and ChIP-seq data. Our algorithm rediscovers experimentally verified disease-associated CRs (polydactyly diseases) that alter gene expression by disrupting chromosome 3D structure. Interestingly, we find that intellectual disability may be a candidate disease caused by 3D chromosome structure alteration. We also develop a Web server (3Disease Browser, http://3dgb.cbi.pku.edu.cn/disease/) for integrating and visualizing disease-associated CR events and chromosomal 3D structure.

Chromosomal rearrangement (CR) events are observed in many tumor and non-tumor human diseases^1^,^2^. A CR event occurs as a consequence of double-strand breaks (DSBs) of DNA, followed by abnormal rejoining of non-homologous ends, producing a new chromosomal arrangement^3^. Alternatively, a CR event results from crossing-over between repetitive DNA sequences during cell division^4^. CR events are categorized into different types: deletion, duplication, insertion, inversion, translocation and ring chromosome. The information of CR events and their associated diseases and clinical symptoms is available in databases such as ClinVar^5^ and dbCRID^6^.

CRs have functional consequences in gene regulation and expression. CRs may result in the formation of a fusion gene from which a fusion transcript and protein is generated. An example is the BCR-ABL gene in chronic myeloid leukemia (CML)^7^. The characteristic genetic abnormality of CML is a reciprocal translocation between the long arms of chromosomes 9 and 22^7^. The molecular consequence of this translocation is the generation of the fusion protein BCR-ABL, a constitutively activated tyrosine kinase. A drug specifically targeting this fusion protein proved effective for CML patients^8^.

Chromosomes in the nucleus fold into highly complex three-dimensional structures that are dynamically regulated^9^,^10^,^11^,^12^. 3C-based techniques such as 5C, Hi-C and ChIA-PET capture genome-wide spatial interaction between chromatins at a high resolution^13^,^14^,^15^. The generated data result in deeper insights into the 3D genome and its functional consequences. For example, the interactions between gene promoters and remote enhancers are facilitated by loop structures between them^15^. The mammalian genome is organized into ~1 Mb topologically associated domains (TADs) which are functional units of gene replication and transcription^16^,^17^. When CRs alter chromosomes, they may also alter chromosomal 3D structure^18^,^19^. Figure 1a shows a model of TAD variation caused by CRs. In condition 1, TAD A and TAD B are insulated by a TAD boundary, which is often occupied by the CTCF protein. The enhancer in TAD B cannot interact and activate the gene in TAD A. However, when the TAD boundary is deleted by a CR, TAD A and TAD B merge into a single TAD C. In this condition, the enhancer and the gene can interact with each other, leading to gene activation. A de novo TAD boundary may also be generated by duplication or inversion events. Therefore, the structural variations of TADs can influence gene expression^18^.

In recent years, advances in DNA microarray and sequencing technologies have led to accurate identification of CR events and chromosomal 3D structures in large scale^5^,^13^. Combining these data sets may provide us more insights on the pathogenicity of CR events that affect chromosomal 3D structure. In this study, we predicted disease-associated CR events that may alter chromosomal 3D structure by integrating Hi-C and ChIP-seq data sets. We also developed a Web server for easily exploring the relationship between disease-associated CR events and chromosome 3D structures of multiple human cell types.

## Results

### Predicting disease-associated CR events that affect TADs

**Figure 1:**
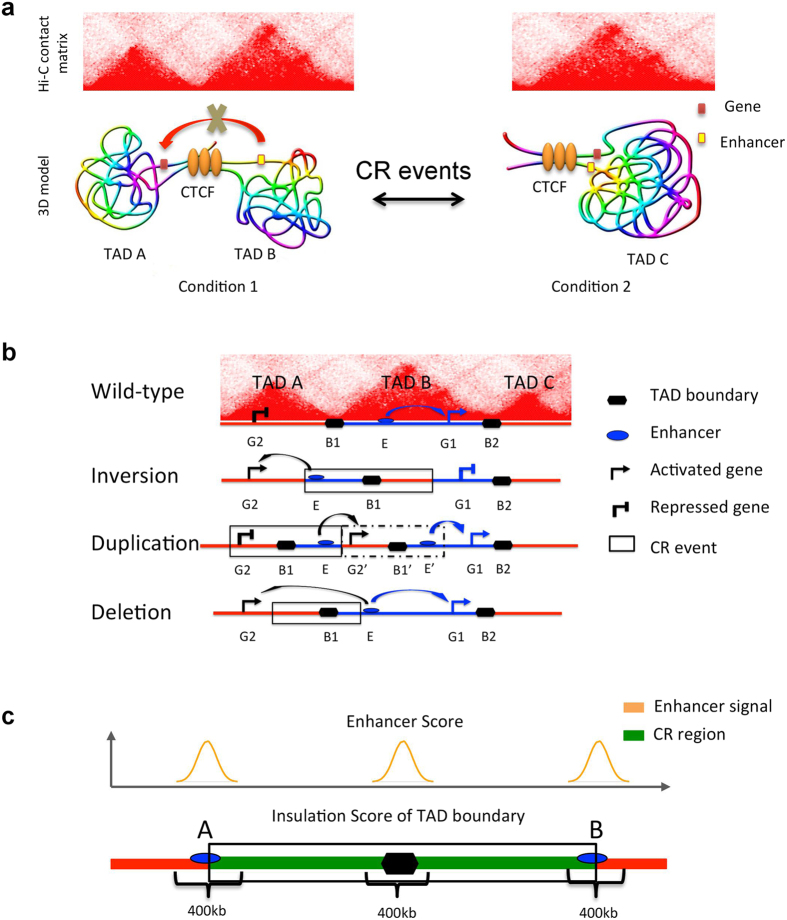
Models for how CR events cause Chromosome 3D structure variation. (**a**) A model of chromatin TAD variation caused by CR. In the Hi-C contact matrix, every red triangle represents one TAD. (**b**) A model for pathogenicity of CR events that alter gene expression through 3D chromosome structure (adapted from ref. 18). (**c**) The scores used to predict disease-associated CR events that affect TADs. SI Score = Percentile (Insulation Score) * Percentile (Enhancer Score).

Based on previous studies, abnormal 3D chromosome structure may be a consequence of CR events leading to diseases. When CR events affect TAD boundaries, they alter the interactions between genes and enhancers, leading to abnormal expression of genes (Fig. 1a). A recent study shows that CR events cause polydactyly diseases through altering 3D chromosomal organization^18^. A model for pathogenicity of CR events is shown in Fig. 1b. Wild-type chromosome conformation shows the structure of three TADs, separated by boundary elements. The activity of an enhancer (E) is restricted to gene 1 (G1) located inside TAD B. In an inversion event, the enhancer is moved out of TAD B and placed near gene 2 (G2), and the boundary is now on the right side of E. This results in interaction between E and G2, but prevents the original interaction between E and G1. In a duplication event, E is placed next to the duplicated G2 within a newly created TAD, resulting in their interaction and abnormal expression of G2 (Fig. 1b). Lastly, if a deletion event removes the boundary B1 and parts of TAD A and TAD B, the two TADs merge and E is able to interact with both G1 and G2, resulting in abnormal expression of G2.

Based on the above model, we calculate an SI (Structure Influence) score, which quantifies the degree that a disease-associated CR alters 3D chromosome structure (Fig. 1c). We inspect two features from these types of CR events: inversion, duplication and deletion. First, a CR region contains at least one TAD boundary, so the CR probably disrupts the structure of neighboring TADs. Second, there must be at least one predicted enhancer near the CR breakpoints or the TAD boundary, so the enhancer may mis-regulate the expression of nearby genes in disrupted TADs. To quantitatively evaluate these two features, for each CR, we calculate an Insulation Score measuring the strength of TAD boundaries in the CR^15^,^20^,^21^, and an Enhancer Score measuring the abundance of nearby enhancers (see Methods, Fig. 1c). The SI score is given by SI = Percentile (Insulations Score) ∗ Percentile (Enhancer Score), measuring the degree that a disease-associated CR may alter gene expression through changing 3D chromosome structure.

### Top predicted disease-associated CR events that affect TADs

**Figure 2:**
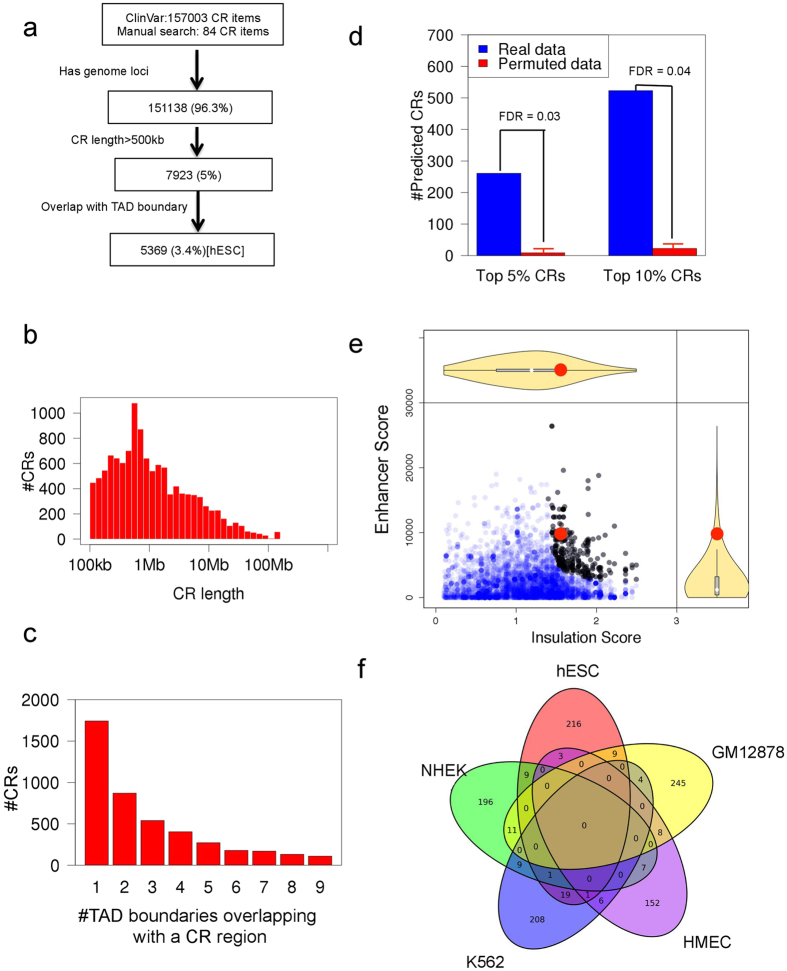
Predicting disease-associated CR events that affect TADs. (**a**) The CR-filtering workflow. (**b**) The length distribution of the filtered CR regions. (**c**) The number of CRs with different number of overlapping TAD boundaries. (**d**) The FDR of the top predicted CRs. (**e**) The scatterplot of Insulation Score vs. Enhancer Score. Black points represent the CR events with top 5% highest SI score. The red point represents a CR associated with polydactyly disease. (**f**) The overlap between top 5% predicted CRs using different cell lines.

We collect 157,003 disease-associated CR events from ClinVar (http://www.clinvar.com)^5^ and 84 additional CRs by manual search in PubMed. We filter the data by genome loci, CR region length and proximity to TAD boundaries in the hESC (human embryonic stem cell) cell line (Fig. 2a), and obtain 5369 disease-associated CR events for the next analysis. The median length of these CR regions is about 800 kb (Fig. 2b). Considering the similar size of the filtered CRs and TADs (median size is 1.1 Mb in the hESC cell line), these CRs are likely to influence the chromosome 3D structure at the TAD level. The number of TAD boundaries in the hESC cell line overlapping with a CR region is mostly one or two (Fig. 2c).

Using the hESC cell line’s Hi-C and ChIP-seq data, we predict 261 CR events (top 5% SI score, FDR = 0.03, Fig. 2d) that may disrupt TAD boundaries and mis-regulate gene expression (black dots in Fig. 2e, top 20 in Table 1). The FDR is 0.03 and 0.04 for top 5% and 10% predicted CRs (Fig. 2d). There is only a moderate correlation between SI Score and CR length (The Pearson correlation is 0.24). We also use the sum of percentile (Insulation Score) and percentile (Enhancer Score) as a score to predict TAD-altering CR events, and the top 5% of predicted CRs are mostly similar (99% overlapping). The SI score of a CR associated with polydactyly diseases is 0.72, and it is ranked within top 4% among all filtered disease-associated CRs (red dot in Fig. 2e). For four of the five CRs from the Lupianez *et al*. study^18^ that are associated with limb developmental disorders, the SI scores are significant (Table 2). Therefore, our algorithm rediscovers experimentally verified disease-associated CRs that alter gene expression by disrupting chromosome 3D structure.

Although the hESC cell line is a suitable surrogate for the 3D genome of key cell types responsible for developmental diseases, we ask how different cell types affect our prediction. We expand analysis by using Hi-C and ChIP-seq data of six cell lines (GM12878, K562, hESC, HUVEC, HMEC and NHEK) representing different cell types. The top 5% predicted CRs in different cell lines are mostly cell line specific (Fig. 2f). Considering the differences and conservation of TADs and enhancers among cell types, it is essential to match a disease and its related cell types in our analysis. The conservation of the 3D structures (loop level) of different human cell lines is about 55% ~ 75% 15, and the conservation of enhancer can be as low as 30% between HeLa and K562 22. However, Hi-C data are currently not available for many cell types, and we do not find suitable Hi-C data for some diseases. To reduce the influence of mismatched cell types and a disease, we average the SI score of six different cell lines to calculate a mean SI score to screen disease-associated CRs. If the mean SI score is high and the FDR is significant, the TAD boundary and enhancer is more conserved among cell types and the result is less likely to be affected by mismatched cell types and disease.

### Visualizing the relationship between TADs and CR events

**Figure 3:**
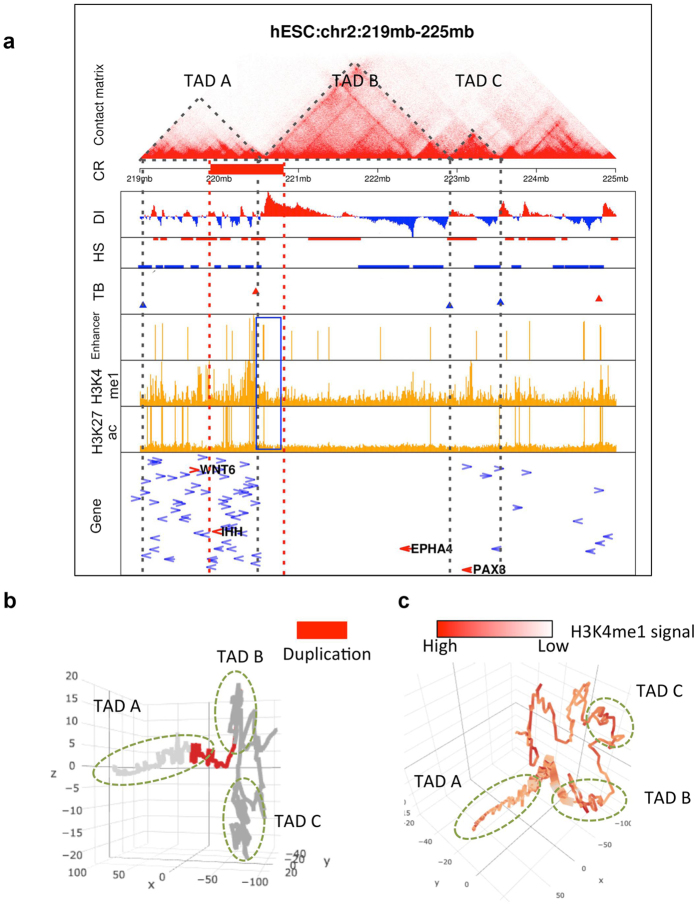
Visualization of the relationship between TADs and a duplication CR associated with polydactyly disease. (**a**) The contact matrix is the heatmap of Hi-C contact frequencies in hESC, with the deeper color for greater value of contacts. The dotted triangles indicate identified TADs. The red bar represents the CR region. DI (Directionality Index), HS (HMM state) and TB (TAD boundary) scores identify TAD boundaries. The enhancer row shows the number of “strong enhancer” base-pairs in 10 kb bins. The H3K4me1 and H3K27ac ChIP-seq data confirm the enhancers. The last row is gene information, including the gene name, length and transcription direction. (**b**,**c**) The 3D model of the TADs surrounding the duplication region. The red bar in b and thick bar in c represent the CR region.

We make several types of graphs to help us understand how CR events may lead to diseases via altering 3D chromosome structure. The first graph contains detail information about TADs, including Hi-C contact matrix, TAD boundaries^16^,^21^, ChIP-seq data and gene information (Fig. 3a). The second graph shows the 3D model of the chromosome structure surrounding a CR event (Fig. 3b). The third graph is the 3D model of the chromosome structure overlaid with additional genomic annotations (Fig. 3c, H3K4me1). These graphs help us to understand how CR affects 3D chromosomal structure and the distribution of enhancer on the 3D structure. Here we show two examples using known and predicted TAD-affecting CRs. For a CR of a patient with polydactyly disease^18^, the SI score (hESC) and mean SI score are both significant: 0.72 (FDR = 1.1%) and 0.23 (FDR = 1.1%), respectively. In this patient, there is a 926 kb duplication (red bar in Fig. 3a) in chromosome 2. This duplication causes an enhancer (blue box) ectopically interact with the nearby gene IHH, leading to high expression of IHH^18^. A specific spatial and temporal expression of IHH within the developing limb buds is essential for accurate digit outgrowth and correct digit number^23^. The 3D models of the TAD regions surrounding the duplicated region show that the duplication affects both TAD A and TAD B (Fig. 3b,c).

**Figure 4:**
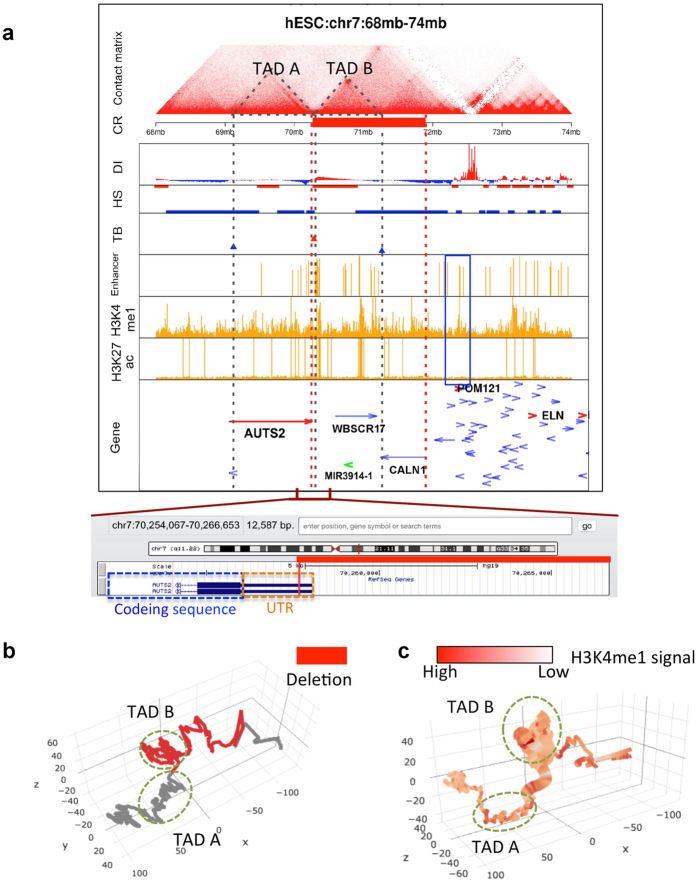
Visualization of the relationship between TADs and a deletion CR associated with intellectual disability disease. (**a**) Similar plot to Fig. 3a for a deletion CR. The detailed information of the deletion region and nearby genes is added to the bottom. (**b,c**) The 3D model of the TADs surrounding the deletion region. The red bar in b and thick bar in c represent the CR region.

**Figure 5:**
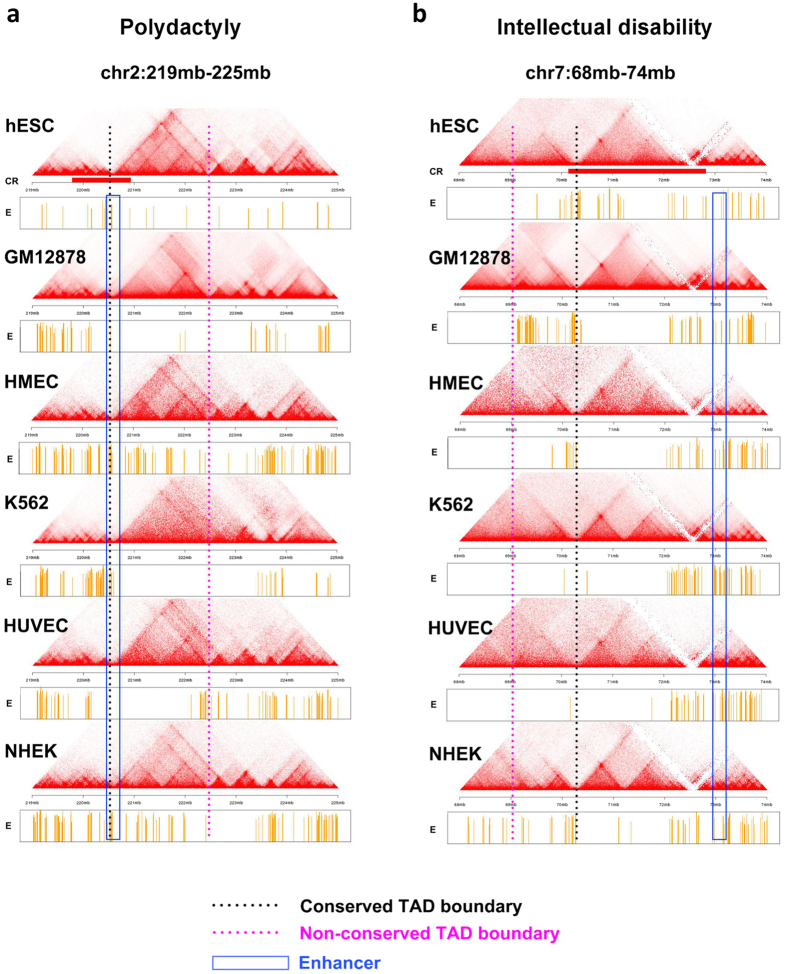
Differences and conservation of TADs and enhancers among cell types. (**a**) The contact matrix and enhancers near a CR associated with polydactyly in six cell lines. The blue box indicates the enhancer region that contributes to the Enhancer score. The black dotted line is the TAD boundary that contributes to the Insulation score. (**b**) Similar plot to a for a CR associated with intellectual disability.

Among our top predicted CR events, one is associated with intellectual disability^2^. The SI score (hESC) and mean SI score of this CR are both significant: 0.40 (FDR = 4.7%) and 0.11 (FDR = 4.7%), respectively. In this patient of intellectual disability, there is a 1.6 Mb deletion (chr7: 70,257,735 - 719,093,76, red bar in Fig. 4a)^24^. The deleted genes (MIR3914-1, WBSCR17 and CALN1) are not known to relate to intellectual disability. At one side of the deletion is the gene AUTS2 (Autism susceptibility candidate 2, 7q11.22). The deletion in this patient does not affect the coding region of AUTS2 (Fig. 4a). Nevertheless, previous studies show that genomic rearrangements involving AUTS2 are associated with autism and intellectual disability^2^. In some patients CR events alter AUTS2’s coding region, and in other patients disruption of intergenic regions may drive the phenotype^2^. In particular, abnormal expression of the AUTS2 may cause phenotypes of intellectual disability^25^. Since AUTS2 locates within one TAD (Fig. 4a), when the deletion removes the TAD boundaries near its one end, nearby enhancer elements (blue box) may alter the expression of AUTS2. Hence we propose that the deletion may affect the expression of AUTS2 through changing the local 3D chromosome structure (Fig. 4b,c). In both examples, the TAD structures and enhancers are largely conserved among different cell types (Fig. 5), leading to significant mean SI scores.

### Web server for the 3D structure of disease-associated CR events

We implement a constraint-based modeling that converts Hi-C data to 3D chromosome models based on the ShRec3D algorithm^26^. It uses a shortest-path method to convert Hi-C contact frequencies to relative distances between chromosome bins, and then uses the multi-dimensional scaling (MDS) method to reconstruct the three-dimensional coordinates of the bins that fit to their pair-wise distances. By using this workflow, we convert the Hi-C interaction matrix surrounding a CR region to 3D models (Figs 3b and 4b).

**Figure 6:**
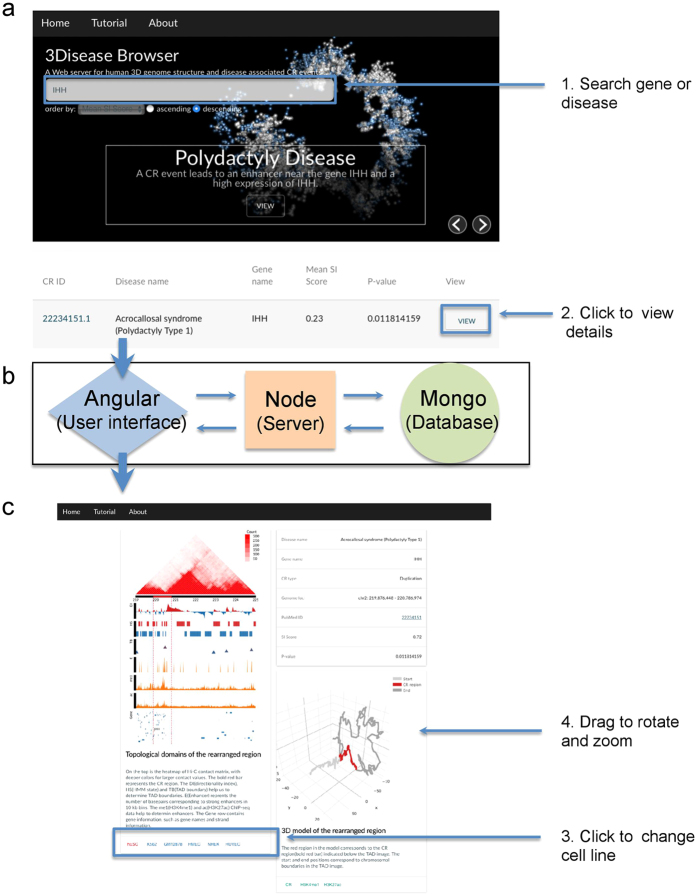
The 3Disease Browser Web server. (**a**) The home page of the 3Disease Browser with a demonstration example of the IHH gene. The input parameters are CR ID, disease name or gene name. (**b**) The internal design of the Web server. (**c**) The TAD and 3D visualization of the queried CR region, similar to Figs 3 and 4.

To display and share the integrated data and prediction results, we developed a Web server for querying and visualizing disease-associated CR events and 3D genome data (3Disease Browser, http://3dgb.cbi.pku.edu.cn/disease/). The visualization of the disease-associated CR and Hi-C data is user-friendly: (1) Type in an interested gene name or disease name in the search panel, and the records containing the query texts will be displayed below the search box (Fig. 6a). (2) Click the “View” link for a particular item in the list and it will generate a new page with detailed TAD and 3D model information for the particular item (Fig. 6c). (3) The default cell line is hESC, and click a different cell line name to view the corresponding data for that cell line. (4) Use mouse to interactively rotate and zoom the 3D structure, and click different ChIP-seq data buttons to view the corresponding epigenetic information overlaid on the 3D structure.

## Discussion

CR events are frequently associated with cancer and developmental diseases. In addition to their roles in disrupting coding regions, recent studies found that CRs may alter gene expression by affecting chromosomal structures. In this study, we developed a method to predict disease-associated CRs that may influence chromosomal 3D structure using Hi-C and ChIP-seq data. Our method rediscovers experimentally validated disease-causing CRs in the polydactyly diseases that alter gene expression by disrupting chromosome 3D structure^18^. Among our top predicted CR events, intellectual disability is a candidate disease caused by TAD-affecting CRs. This new discovery provides important clues about the causes of intellectual disability. Follow-up validations may compare the gene expression level of normal people and patients with CR in affected cell types, and use 4C and CRISPR experiments to inspect the impact of CRs on chromatin interactions.

In addition to TADs, chromatin loops are identified by 3D genome data^15^. Mammalian genomes are partitioned into larger TAD domains that contain smaller chromatin loops linking promoters and enhancers^27^,^28^. Capture Hi-C is used to investigate the interactions between autoimmune diseases-associated SNPs and genes^29^. In the future, we will expand our method to predict chromatin loops that are disrupted by disease-associated CRs or SNPs such as those in cancer^30^. Other ChIP-seq markers such as those indicating promoters may also be added to the scoring algorithm.

Although the 3D structures of different cell lines are conserved^15^, the enhancer patterns in different cell lines are cell line-specific^22^, causing differential TAD-disrupting capacity of a CR in different cell types. Therefore, TAD-affecting CR prediction should match a disease and its related cell types. We use mean SI score to mitigate this issue. Recently the chromatin interaction maps of 98 cell lines were predicted from histone modification data^31^. For future work, we will add these data to our model and Web server to better match diseases and 3D genome data.

We develop 3Disease Browser (http://3dgb.cbi.pku.edu.cn/disease/) for querying and visualizing human chromosomal 3D structure and disease-associated CRs. Existing 3D genome browsers can display Hi-C contact matrices (Hi-C Data Browser, Dekker lab), overlay annotation tracks such as ChIP-seq data and genes with the Hi-C heatmaps (Juicebox, Lieberman- Aiden lab), and visualize 3D genome structure (GMOL). Recently, the HiView Web server combines the GWAS variants with Hi-C data^32^. Our 3Disease Browser is the first disease-centric 3D genome browser that integrates Hi-C data, annotation tracks and 3D structure overlaid with epigenetic information (Table 3 for a comparison of 3D genome browsers).

In summary, integrating data of chromosomal 3D structure and disease-associated CRs provides important clues about disease pathogenicity. We made a first attempt to systematically correlate these data types to prioritize disease-related genomic alterations that may contribute to disease initiation and progression through 3D genome reorganization. Our methods and Web server are easily expandable to integrate other data types with 3D genome data, such as copy number alterations in cancer and disease-associated loci from GWAS studies.

## Methods

### Data sets

We used high-resolution Hi-C data from Gene Expression Omnibus (GEO) with the accession number GSE63525 (GM12878, K562, HMEC, HUVEC and NHEK)^15^ and GSE35156 (hESC)^16^. The current version of 3Disease Browser contains these six human cell types (http://genome.ucsc.edu/ENCODE/cellTypes.html). GM12878 is a lymphoblastoid cell line produced from the blood of a female with European ancestry. K562 is an immortalized cell line from a female patient with chronic myelogenous leukemia (CML). hESC is an embryonic stem cell line from a normal male. HMEC is a mammary epithelial cell line from a normal person. HUVEC is an umbilical vein endothelial cell line from a normal person. NHEK is an epidermal keratinocyte cell line from the skin of a normal person. The ChIP-seq data of the above cell lines come from the ENCODE project (https://www.encodeproject.org). The CR data come from ClinVar (Release 2015-12, http://www.ncbi.nlm.nih.gov/clinvar/) and manual search of PubMed.

### Calculation of Structure Influence score

We calculated the Insulation score^15^,^20^,^21^ of each chromosomal bin using the 10 kb resolution Hi-C data. DI (Directionality Index) and HS (HMM State) were calculated using the Dixon method^16^. The Insulation Score of a CR region is the highest score of the TAD boundaries identified in the CR. The enhancer data come from the Broad ChromHMM track in the UCSC Table Browser (http://genome.ucsc.edu). We only used the strong enhancer (state 4 and state 5) to calculate the Enhancer Score. For a CR event, we calculated the sum of enhancer base pairs in each of the three 400 kb regions surrounding the two CR breakpoints and the TAD boundary with the highest insulation score TAD boundary as the Enhancer Score. Finally we calculated the SI score as Percentile (Insulation Score) * Percentile (Enhancer Score), with percentile relative to the respective scores of other CR regions. Alternatively, we also calculate the sum of enhancer base pairs of the whole CR region normalized by CR length as the Enhancer Score (Figure S1a). The Pearson’s correlation of the two methods’ SI score is high (0.87, Figure S1b). We conclude that both methods have their merits.

To calculate the FDR of top 5% scores, we permuted corresponding relationship between Insulation Score and Enhancer Score of the CR regions 1000 times. Each time, we record the number of SI scores larger than 0.68 (the smallest score of top 5%). The ratio between the mean number of SI scores > 0.68 in permuted data and the number of top 5% CRs is the estimated FDR. The minimum FDR of the six cell lines is regard as the FDR of mean SI score.

### Web server implementation

3Disease Browser is built by using the MongoDB/ExpressJS/AngularJS/NodeJS stack software. It uses D3.js for interactive 2D presentation in SVG layers and Plotly.js for 3D visualization. jQuery is used for writing Javascript. It uses queue.js for asynchronous data loading and Masonry for cascading grid layout.

## Additional Information

**How to cite this article**: Li, R. *et al*. 3Disease Browser: A Web server for integrating 3D genome and disease-associated chromosome rearrangement data. *Sci. Rep.* **6**, 34651; doi: 10.1038/srep34651 (2016).

## Supplementary Material

Supplementary Information

## Figures and Tables

**Table 1:** Top 20 diseases whose associated CRs are predicted to affect TAD.

CR ID	Disease Name	CR Type	CR length	Gene	PubMed ID	SI Score (hESC)	FDR (SI score)
145620	Cleft palate	Deletion	3630934	CRYBB2	20466091	0.91	0.04%
145534	Anal atresia	Duplication	933288	GMDS-AS1	20466091	0.84	0.23%
59058	Arthrogryposis multiplex congenita	Duplication	840627	APOL4	21844811	0.84	0.25%
33260	Hearing impairment	Deletion	2641573	CLDN5	20466091	0.84	0.25%
59358	Attention deficit hyperactivity disorder	Deletion	2158318	INADL	21844811	0.83	0.29%
57809	Proteinuria	Deletion	4121281	AHSA1	21844811	0.83	0.29%
58696	Total anomalous pulmonary venous return	Duplication	6350820	KRT39	21844811	0.81	0.39%
155396	Tracheomalacia	Deletion	1865737	RGS14	20466091	0.80	0.54%
57591	Growth delay	Deletion	7912990	RFX4	21844811	0.79	0.58%
60244	Arachnoid cyst	Deletion	9625433	GTF2IRD2	21844811	0.78	0.65%
57341	Epispadias	Duplication	747247	MIR1243	21844811	0.77	0.80%
155202	Leukodystrophy	Deletion	1058291	GJA9-MYCBP	20466091	0.75	0.95%
153687	Sepsis	Deletion	5756016	DDX41	20466091	0.74	1.02%
57240	Ambiguous genitalia	Duplication	1500151	ATL2	21844811	0.72	1.16%
22234151	Polydactyly type 1	Duplication	926183	IHH	22234151	0.72	1.16%
25959774.1	F-syndrome Type 2	Inversion	1098179	WNT6	25959774	0.72	1.16%
18272352	Polydactyly	Deletion	623734	EPHA4	18272352	0.72	1.16%
57202	Pneumonia	Duplication	2914266	DGCR6	21844811	0.72	1.16%
33463	Microcephaly	Deletion	2734514	TXNRD2	20466091	0.72	1.16%
144137	Spina bifida occulta	Deletion	2785959	TTC3-AS1	20466091	0.70	1.43%

CR ID: ID in 3Disease Browser. Gene is the candidate gene name affected by CR events. SI Score (hESC) is calculated using hESC cell line’s data.

**Table 2:** Five CRs associated with limb developmental disorders.

CR ID	Disease Name	CR Type	Genome Loci	Gene	SI Score (hESC)	FDR (SI score)
25959774.3	Brachydactyly	Deletion	Chr2:221278232-223014332	PAX3	0.017	38.7%
25959774.1	F-syndrome	Inversion	Chr2:219741632-220839811	WNT6	0.724	1.1%
25959774.2	F-syndrome	Duplication	Chr2:219295343-220501540	WNT6	0.724	1.1%
22234151	Polydactyly	Duplication	Chr2:219876448-220786974	IHH	0.724	1.1%
18272352	Polydactyly	Deletion	Chr2:220019492-220643226	IHH	0.724	1.1%

CR ID: ID in 3Disease Browser. SI Score (hESC) is calculated using hESC cell line’s data.

**Table 3:** Comparison of 3D genome browsers.

Web server or software	Hi-C heatmap display	Annotation tracks	Gene search	Disease search	GWAS variants search	3D model display	PubMed ID	Website
3Disease Browser	✓	✓	✓	✓		✓	—	http://3dgb.cbi.pku.edu.cn/disease
ChromContact		✓	✓		✓		26666652	http://bioinfo.sls.kyushu-u.ac.jp/chromcontact/
HiView		✓	✓	✓	✓		26969411	http://www.unc.edu/~yunmli/HiView/
Hi-C data Browser (Dekker lab)	✓						—	http://hic.umassmed.edu/welcome/welcome.php
HiC-3DViewer	✓	✓				✓	—	http://bioinfo.au.tsinghua.edu.cn/member/nadhir/HiC3DViewer
Hi-C data Browser (Yue lab)	✓	✓					—	http://yuelab.org/hi-c/database.php
WashU EpiGenome Browser (Wang lab)	✓	✓	✓		✓		23629413	http://vizhub.wustl.edu/
Juicebox (Lieberman- Aiden lab)	✓	✓					25497547	http://www.aidenlab.org/juicebox/
4DGenome Browser		✓	✓				25788621	http://4dgenome.int-med.uiowa.edu
Genomic HyperBrowser		✓					21182759	https://hyperbrowser.uio.no/hb/
GMOL						✓	26868282	https://sourceforge.net/projects/gmol/
Genome3D						✓	25348407	http://www.genome3d.eu
Tadkit		✓				✓	—	http://sgt.cnag.cat/3dg/tadkit/
