# Supplementary material for: 3Disease Browser: A Web server for integrating 3D genome and disease-associated chromosome rearrangement data
Source: Sci Rep. 2016 Oct 13;6:34651. doi: 10.1038/srep34651 (PMC5062081; doi:10.1038/srep34651)
Supplement: Supplementary Information [file srep34651-s1.doc]

**Supplementary information**

**3Disease Browser: A** **Web server for integrating 3D genome and disease-associated chromosome rearrangement data**

Ruifeng Li^1,#^, Yifang Liu^2,#^, Tingting Li^1^, Cheng Li^1,3,*^

1. Peking-Tsinghua Center for Life Sciences, Academy for Advanced Interdisciplinary Studies; School of Life Sciences, Peking University, Beijing, China
2. School of Life Sciences, Tsinghua University, Beijing, China
3. Center for Statistical Science; Center for Bioinformatics, Peking University, Beijing, China

# Equal contribution

* Correspondence: cheng_li@pku.edu.cn

**Figure S1**

**Figure S1. An alternative Enhancer score used to predict disease-associated CR events that affect TADs.**

1. The scoring model. The CR region is extended 200 kb at both sides

SI Score = Percentile(Insulation Score) * Percentile(Enhancer Score)

Enhancer Score = Sum of Enhancer base pairs of the extended CR region / length of the extended CR region

**b.** The scatterplot of SI Score using enhancer data of the whole CR vs. SI Score using only the enhancer data of TAD boundaries and CR breakpoints (hESC).
